# Framing sustainability in Milan’s urban policies: new coalitions in a molecular transition

**DOI:** 10.3389/fsoc.2026.1892885

**Published:** 2026-08-17

**Authors:** Monica Bernardi, Carlos Manzano, Adriano Cancellieri, Simone Tosi

**Affiliations:** Department of Sociology and Social Research, University of Milan-Bicocca, Milan, Italy

**Keywords:** cities, Milan, policy, socio-ecological change, sustainability, transition, urban coalitions, urban environmental governance

## Abstract

This article argues that socio-ecological transition in Italian cities unfolds less as a coherent policy trajectory and more as a fragmented and contested field of governance devices, discourses, and coalitions. Drawing on a systematic mapping of sustainability-related policy instruments adopted in Milan between 2015 and 2025, discourse analysis, and key-informant interviews, Milan is approached as a critical urban laboratory. The paper shows how sustainability is produced through a proliferation of partially autonomous policy instruments, what we conceptualise as *sustainability molecules*, whose alignment is periodically attempted around major *turning points* such as mega-events, crises, and political cycles. These moments typically do not produce systemic integration; instead, they tend to reconfigure urban coalitions and legitimise selective interpretations of sustainability, highlighting its dual role as both a transformative agenda and a dispositif of urban legitimation.

## 1 Fragmented transitions: an introduction

Over the last decade, cities have been increasingly positioned as key arenas for socio-ecological transition (Bulkeley and Betsill, 2003). From climate mitigation and adaptation to biodiversity protection, circular economy strategies, and sustainable mobility, urban policies are now widely framed as the primary vehicles through which environmental transformation can be accelerated and made tangible in everyday life. In both policy discourse and academic debate, cities are frequently portrayed as laboratories of sustainability innovation, expected to compensate for the inertia or fragmentation of national and international governance (Bulkeley and Betsill, 2005; Bulkeley et al., 2014; Acuto et al., 2018; UN-Habitat, 2020). Within this narrative, urban transition is often imagined as a directional and cumulative process in which policies, actors, and instruments progressively align around shared environmental goals (Geels and Schot, 2007; Markard et al., 2012). Nevertheless, empirical observation suggests a more ambivalent picture. In practice, socio-ecological transition in cities rarely unfolds as a coherent strategy or an integrated governance architecture. More often it emerges through a dense and heterogeneous assemblage of plans, programmes, regulatory tools, partnerships, pilot projects, co-design and participatory arenas, often developed in response to specific political windows, funding opportunities, crises, or flagship events (Bulkeley and Castán Broto, 2013; Hajer et al., 2015). These instruments coexist, overlap, and sometimes contradict one another, reflecting divergent interpretations of sustainability and shifting constellations of power (Swyngedouw, 2007; While et al., 2004). In this sense, urban transition does not produce a linear pathway towards transformation, and more often it takes the form of an unstable field of governance, where meanings, priorities, networks and legitimate forms of action remain contested (Davoudi et al., 2013; Whitehead, 2013).

This tension between the promise of transition and its fragmented governance is particularly visible in the context of Italian cities. On the one hand, Italian urban centres have increasingly embraced the language of sustainability, climate action, and resilience, aligning themselves with international agendas, transnational networks, and European policy frameworks (European Commission, 2019; OECD, 2020). On the other hand, Italian urban political economy tradition deals with how sustainability is mobilised within urban governance (Vicari and Anselmi, 2020; Cucca, 2012). As a result, socio-ecological transition in Italian cities often appears less as a systemic reorientation and more as a selective, negotiated, and sometimes even opportunistic process.

The article does not treat this condition as a sign of policy failure or institutional lag, instead, it approaches Italian cities as privileged sites for a critical assessment of socio-ecological change. The article argues that the Italian urban context makes visible dynamics that are often less explicit in many contemporary cities: the coexistence of transformative ambitions and the legitimising functions of sustainability; the reliance on fragmented governance devices rather than unified transition strategies; and the central role of discourse in stabilising temporary alignments among heterogeneous actors (Harvey, 1989; Whitehead, 2013; Conte and Anselmi, 2022). In this sense, Italian cities do not simply “lag behind” more celebrated transition frontrunners, they expose the political and discursive work through which urban sustainability is continuously assembled, negotiated, and stabilised through multiple governance instruments.

Within this broader landscape, Milan represents a particularly revealing empirical laboratory. Over the past decade, the city has combined an intensified international projection with deep transformations of its urban development model. Key conjunctural moments, such as the EXPO 2015 legacy, the declaration of a climate emergency in 2019, the COVID-19 pandemic, and the preparation of the Milano-Cortina 2026 Winter Olympics have all acted as powerful conjunctures shaping the city’s sustainability agenda. Across these moments environmental concerns have become increasingly central to urban policy, while simultaneously being entangled with competitiveness, territorial branding, real estate development, and infrastructural investment (Harvey, 1989; Theodore et al., 2011).

The result is a proliferation of sustainability-related policy instruments, including climate and air plans, mobility strategies, food policies, regenerative programmes, participatory processes, and event-related sustainability frameworks. All these instruments have formed a dense and heterogeneous policy landscape, characterised by multiple temporalities, actor constellations, and justificatory repertoires. Milan’s transition thus appears not as a unified project, but as a field of partial alignments and ongoing tensions over what sustainability should mean, how it should be governed, and whose interests it should serve.

This article starts from the premise that making sense of Milan’s socio-ecological transition calls for an approach that goes beyond outcome-centred evaluations and diagnoses of implementation gaps. It examines the transition as a discursive and coalition-building process in which sustainability functions both as an agenda for environmental transformation and as a Foucauldian *dispositif* of urban legitimation, that is a configuration of discourses, institutional arrangements, policy practices, and governing rationalities through which specific urban development trajectories are rendered legitimate. This perspective draws on critical urban theory and urban political economy, which have long highlighted how environmental policies can stabilise entrepreneurial and financialised modes of governance while deferring deeper structural change (Harvey, 1989; Whitehead, 2013; While et al., 2004). At the same time, it engages with scholarship on urban governance and regimes, emphasising the role of actor coalitions, narrative alignment, and institutional experimentation in shaping urban change (Stone, 1989; Davies, 2011; Hajer, 1997).

To capture these dynamics the article mobilises an interpretative framework centred on *frame analysis* (Snow and Benford, 1992; Benford and Snow, 2000) where frames are understood as organising principles that structure how problems are defined, responsibilities assigned, solutions legitimised. In the context of Urban Environmental Governance (UEG), frames do not merely circulate at the level of discourse, but they become embedded in policy instruments, indicators, participatory formats, partnership arrangements, acquiring performative effects (Hajer, 2003; Kitchin, 2014). Analysing frames therefore allows us to connect symbolic constructions of sustainability to the concrete devices through which it is governed.

Building on this perspective, the article introduces the concept of *sustainability molecules* to capture how socio-ecological transition in Milan is governed through a proliferating set of policy instruments that operate as partially autonomous yet relationally connected units, interacting and occasionally aligning around specific political and institutional *turning points*, such as mega-events and crises. These function as powerful discursive catalysts that open policy windows, accelerate decision-making, and intensify the circulation of desirable futures (Kingdon, 1995; Leach et al., 2010). Yet, these same conjunctures can amplify tensions between sustainability as a collective right and sustainability as an urban asset, between systemic-ecological understandings of the city and technocratic reductions of complexity into targets and performance indicators (Checker, 2020; Conte and Anselmi, 2022; Bibri, 2019). The analytical task, then, is to reconstruct how these tensions are articulated across the city’s *sustainability molecules*, how frame alignment occurs (or fails), and how shifting coalitions consolidate particular interpretations of sustainability at key historical moments.

Empirically, the study combines a systematic mapping of Milan’s sustainability-related policy instruments between 2015 and 2025 with an in-depth discursive analysis of a selected corpus of strategic documents and semi-structured interviews with key urban actors. This design makes it possible to trace how *environmental sustainability frames* circulate across governance devices, how coalitions are formed and reconfigured, and how meanings shift in relation to political cycles and conjunctural events.

In terms of structure, the article is divided into five sections. Section 2 reconstructs the conceptual background and outlines the analytical approach adopted, bringing into dialogue the literature on sustainability transitions, critical urban political economy, and frame analysis to conceptualize urban socio-ecological governance as a fragmented, relational, and politically contested field. Section 3 presents the research design, sources and methods, detailing the mapping of sustainability-related governance instruments in Milan between 2015 and 2025, the selected documentary corpus for discourse analysis, and the semi-structured interviews conducted with key urban actors. Section 4 develops the empirical analysis and main findings. It first identifies the main turning points that have shaped the evolution of Milan’s socio-ecological governance; it examines how policy instruments, coalitions, and policy priorities have progressively layered and stratified over time; it then identifies and analyses the dominant discursive frames and sense-making processes within UEG devices. Finally, section 5 discusses these findings in relation to the broader debate on urban transitions, arguing that fragmentation should be understood not merely as a coordination deficit but as a specific mode of governing socio-ecological change. Results position Milan not simply as a local case but as a revealing vantage point from which to critically interrogate the possibilities, limits, and contradictions through which socio-ecological transition is unfolding in contemporary Italian cities.

## 2 Fragmentation, power, and urban sustainability: theory and conceptual background

A substantial strand of sustainability transitions scholarship has conceptualised socio-ecological change through the language of pathways and trajectories, understood as patterned sequences of interactions across niches, regimes, and broader socio-technical landscapes that gradually reconfigure dominant systems (Geels and Schot, 2007; Markard et al., 2012). Within this framework, transitions are typically analysed as long-term and directional processes, where coordination, alignment, and cumulative learning play a central role. This vocabulary has proved particularly productive in domains such as energy and mobility, where relatively bounded systems and identifiable technological regimes lend themselves more readily to pathway-based analysis (Geels, 2019).

When applied to urban contexts, however, this perspective reveals important limitations. Cities rarely operate through a single steering centre or a clearly delineated regime. Urban governance is instead characterised by fragmented authority, overlapping competences, and strong exposure to political, economic, and symbolic pressures that are unevenly distributed in space and time (Bulkeley and Betsill, 2005; Lawhon and Murphy, 2012; Bernardi, 2026). These conditions favour forms of governance in which multiple centres of decision-making coexist and policy change unfolds through the accumulation and recombination of instruments over time, rather than through coordinated shifts or institutional replacement (Ostrom, 2010; Mahoney and Thelen, 2010). In this sense, urban sustainability transitions often appear less as orderly trajectories than as contested and spatially differentiated processes, shaped by shifting priorities and competing interpretations of what sustainability should entail.

A growing body of critical work has therefore argued for bringing questions of power, politics, and spatial specificity to the forefront of transition analysis (Smith et al., 2010; Avelino et al., 2016). From this perspective, fragmentation is not simply an implementation gap or a temporary coordination failure. It can instead be read as an empirical condition that reflects how sustainability is governed under contemporary urban political economies. In cities marked by entrepreneurial governance, inter-urban competition, and event-led development, sustainability tends to be mobilised through multiple, partially disconnected instruments, each responding to different agendas and institutional logics, and only intermittently aligned around shared objectives (Harvey, 1989; While et al., 2004).

Urban climate governance scholarship has been particularly attentive to these dynamics. Cities are embedded in complex multi-level constellations of authority and resources, where climate action is shaped by transnational networks, policy mobility, and circulating standards and benchmarks (Bulkeley and Castán Broto, 2013). This perspective resonates with scholarship on policy assemblages and policy mobility, which has highlighted how urban policies circulate, recombine, and stabilise across contexts rather than following linear trajectories (McCann and Ward, 2011; Peck and Theodore, 2015). Governing climate change increasingly takes place through experimentation, such as pilot projects, contracts, indicators, and monitoring infrastructures that translate broad goals into actionable programmes. This translation however is not a neutral process, it tends to privilege what is measurable, comparable, and administratively manageable (Porter, 1995), encouraging technocratic imaginaries of governability and often narrowing the space for political contestation and alternative problem framings (Hajer, 2003; Kitchin, 2014). Long and Rice (2019) frame this shift as the rise of “climate urbanism,” in which urban climate action is increasingly channelled into what is measurable, administratively governable, and readily valorised as investment, especially through carbon management and climate-resilient infrastructures, marking a shift from the broader, more proactive ambitions of sustainable urbanism toward a more selective and reactive agenda that can narrow political contestation and sideline social and redistributive priorities.

These governance dynamics are closely intertwined with longer-standing transformations analysed within critical urban political economy. Since the late twentieth century, urban governance has been increasingly oriented towards competitiveness, place marketing, and investment attraction (Harvey, 1989). Within this context, sustainability frequently operates as a strategic resource: a flexible and malleable signifier that can support growth coalitions, enhance reputational capital, and legitimise selective environmental interventions without necessarily challenging underlying development trajectories. The notion of an “urban sustainability fix” captures this ambivalence, highlighting how environmental policies may stabilise entrepreneurial urbanism while postponing more structural socio-ecological reconfigurations (While et al., 2010; Whitehead, 2013).

At the same time, critical urban theory has drawn attention to the risk of depoliticisation (de Nardis, 2017). Environmental issues are often reframed as matters of technical management, insulated from conflict over distribution, justice, and competing visions of urban futures (Swyngedouw, 2010; Beretta and Bracchi, 2023). From this angle, sustainability is less a consensual policy objective than a contested claim, whose meanings and priorities are continuously negotiated. In urban settings, these negotiations tend to crystallise around tensions between justice- and commons-oriented interpretations, on the one hand, and asset-based, market-oriented framings, on the other (Agyeman, 2013; Foster and Iaione, 2015; Whitehead, 2013; Anguelovski et al., 2018).

To analyse how such tensions are articulated and stabilised, this article adopts an interpretive approach centred on frame analysis. Following Snow and Benford (1992) and Benford and Snow (2000), frames are understood as organising principles that structure problem definitions, allocate responsibilities, and legitimise particular courses of action. In the field of UEG, frames do not circulate only at the level of discourse, they become embedded in policy instruments, indicators, participatory formats, and partnership arrangements, acquiring performative effects and shaping how sustainability is governed in practice (Hajer, 1997, 2003). In fragmented governance settings, framing processes thus constitute a key mechanism through which authority is exercised and coalitions are provisionally stabilised. This perspective also aligns with scholarship on policy instrumentation, which conceptualises policy instruments not as neutral tools but as devices that structure power relations and modes of governing (Lascoumes and Le Galès, 2007).

Building on this interpretive lens, we introduce the concept of *sustainability molecules* to capture how socio-ecological transitions are governed through a proliferating, loosely connected set of sustainability-related policy instruments. A *sustainability molecule* is a policy instrument -a plan, a programme, an agreement, a participatory arena, a regulatory device - understood as a configuration of the meanings, actors, resources, and temporalities that sustain it. Each *molecule* combines: an interpretive framing structure through which environmental problems and legitimate solutions are defined, an actor constellation involved in its design and implementation, an evidentiary regime based on indicators, targets, monitoring procedures, and reporting requirements, and a temporal-financial horizon that shapes its implementation trajectory. We retain the term “*molecule*” instead of instrument deliberately, since it foregrounds the composition of each unit (a compound of heterogeneous elements rather than a single tool), its valence (the uneven capacity to bond with other units through shared actors, narratives, funding streams, and evaluative metrics) and its dynamic repertoire (that is its propensity to combine, stabilise, react, or dissolve under specific political and institutional conditions). Their analytical value lies in making visible both the internal composition of policy instruments and the relations that connect them within a fragmented governance landscape. The concept differs from adjacent notions frequently used in the literature such as “policy instruments” which are commonly understood as the means through which governments pursue specific objectives (Lascoumes and Le Galès, 2007); the notion of *sustainability molecules* shifts attention to their internal composition and relational positioning within a fragmented governance landscape. Or such as the notion of “policy assemblage” which emphasises open-ended and heterogeneous formations that evolve through circulation and recombination (McCann and Ward, 2011); *sustainability molecules* by contrast, remain bounded empirical units that can be systematically mapped and compared. Institutional layering describes the accumulation of instruments over time (Mahoney and Thelen, 2010). *Molecularisation* instead, names the *mode of governing* whereby transition is decomposed into such partially autonomous *sustainability molecules*, together with the selective processes through which some acquire greater visibility, stability, and coordinating capacity than others.

This fragmentation is not merely the outcome of institutional complexity; rather, it reflects the plurality and polysemy of sustainability itself, as well as the diverse ways in which urban actors interpret, appropriate, and mobilise the idea of sustainability. The concept thus helps illuminate how different frames and meanings of sustainability circulate at the discursive level and become embedded in a heterogeneous set of governance devices. Coherence is not an intrinsic property of this configuration; it is an episodic achievement, typically produced under conjunctural pressure and then weakened by further institutional layering.

In line with extended interpretations of policy windows (Kingdon, 1995; Zahariadis, 2007), conjunctural phases in which problem definitions, discursive frames, and governance arrangements are temporarily realigned can be referred as “turning points”. In complex urban governance contexts, such phases tend to generate intensified policy activity and institutional proliferation, often leading to the layering and recombination of policy instruments (Howlett et al., 2015; Mahoney and Thelen, 2010; Boin et al., 2009; Rayner and Howlett, 2009; Jordan and Matt, 2014). Conceptualising sustainability governance as a field structured by *molecularised* policy instruments allows fragmentation to be examined, through the lens of *sustainability molecules*, not simply as a descriptive condition but as an actively produced and negotiated feature of urban transition processes.

This analytical framework informs the empirical strategy of the article, which combines a systematic mapping of sustainability-related governance devices with a frame analysis of selected policy documents and interviews. Together, these tools make it possible to trace how meanings of sustainability circulate across fragmented governance arrangements, how coalitions consolidate around specific framings, and how socio-ecological transition in the city advances through episodic rearticulations.

## 3 Research design: mapping governance devices and sustainability frames

The study employs an exploratory, qualitative multi-method approach. Document analysis (Bowen 2009) and semi-structured interviews were the primary research instruments used to examine processes of *frame alignment* (Snow and Benford, 1992; Benford and Snow, 2000). The analysis focuses on the discourses and coalitions through which “environmental sustainability” becomes legible, actionable, and legitimate within a proliferating ecology of sustainability-related policies and governance devices. More specifically, the research reconstructs how different interpretative frames have shaped the evolution of Milan’s sustainability governance over time and how these frames have contributed to stabilising particular coalitions, policy priorities, and governance devices.

Milan is approached here not as an idiosyncratic exception but as a *critical case* (Flyvbjerg, 2006): precisely because it has long functioned as Italy’s pace-setting “urban laboratory,” the site where instruments of negotiated, public–private, event-driven urbanism have been pioneered before diffusing nationally (Balducci et al., 2017; Lanzani, 2011; Savini, 2014), its sustainability governance offers a magnified view of dynamics that are emerging, more unevenly, across comparable European cities. The very administrative culture that allows Milan to “make things happen” through flexible, reputation-oriented, metric-compatible instruments is what renders its selective treatment of sustainability claims analytically diagnostic and not merely local. Lessons drawn here are therefore not generalisable as a statistical sample, but transferable as an analytically clarified mechanism: how a competitive, growth-oriented urban regime translates a plural idea of sustainability into governable, and unevenly stabilised, *molecules*.

The research design combines an initial mapping of Milan’s main sustainability-related policy instruments and governance devices across the period 2015–2025 with a subsequent policy selection, discourse analysis, and a set of 10 semi-structured interviews.

The initial mapping is guided by a typology that reflects both municipal administrative practices in drafting and managing governance acts and established theoretical frameworks in urban planning and public policy literature. Planning traditionally refers to long-term vision and regulatory instruments (plans, regulations), while programming involves action packages with defined objectives and resources (programs, agreements). Participatory processes represent complementary modes of governance and implementation (Healey, 1997; Arnstein, 1969; European Parliament & Council, 2001; UN-Habitat, 2009). Accordingly, the mapped instruments were categorised across key policy domains, including air and climate, land use, mobility, food and tourism, and urban green spaces and biodiversity.

For comparability and public accessibility, the initial selection focused on official documents available online via the Municipality of Milan’s institutional website.^1^ The systematic review identified 22 sustainability-related policy instruments for the period 2015–2025. The mapping also includes two instruments currently under development and two revised versions of existing policy instruments (see full list in Table 1). The selected instruments are grouped into three categories:

1. Urban and environmental plans and regulations: examples include the Air and Climate Plan (PAC), the Territorial Government Plan (PGT 2030), the Urban Sustainable Mobility Plan (PUMS), the Metropolitan Territorial Plan, and the General Urban Traffic Plan (PGTU).
2. Programs and programming agreements: this category comprises for example the Food Policy, the Program Agreement for Railway Yards, Milano Futura Ora, the Milan Changes Air Program, and the Resilient Neighborhoods Program.
3. Participatory processes and networks: relevant in this category are for instance the Citizens’ Assembly for Climate, and the Milan Air and Climate Alliance.

**Table 1:** Selection of relevant sustainability-related policy instruments for the period 2015–2025.

Policy name	Year	Domain	Category
Milan’s Food policy	2015	Food + Tourism	Programs and programming agreements
Regulation on the Use and Protection of Public and Private Green Spaces	2017	Green + Biodiv.	Urban and environmental plans and regulations
Former railway yards - Program Agreement	2017	Land	Programs and programming agreements
Metropolitan Territorial Plan	2017	Land	Urban and environmental plans and regulations
International Prog. Reinventing cities	2017	Land	Programs and programming agreements
Sustainable Energy Action Plan	2018	Climate + Energy	Urban and environmental plans and regulations
Sustainable Urban Mobility Plan	2018	Mobility	Urban and environmental plans and regulations
Territorial Government Plan (PGT)	2019	Land	Urban and environmental plans and regulations
Open Squares Program	2019	Land	Programs and programming agreements
Regulation on Air Quality	2020	Climate + Energy	Urban and environmental plans and regulations
Milan 2020. Adaptation Strategy	2020	Land	Programs and programming agreements
Citizen Alliance on Climate	2022	Climate + Energy	Participatory processes and networks
Air and Climate Plan - PAC	2022	Climate + Energy	Urban and environmental plans and regulations
Program Milano Cambia Aria	2022	Climate + Energy	Programs and programming agreements
Urban Traffic General Plan (Update.)	2022	Mobility	Urban and environmental plans and regulations
Strategic Guidelines for Biodiversity Management	2023	Green + Biodiv.	Urban and environmental plans and regulations
Resilient Neighbourhoods Program	2023	Land	Programs and programming agreements
Territorial Government Plan (PGT) - (Update)	2023	Land	Urban and environmental plans and regulations
Climate City Contract	2024	Climate + Energy	Participatory processes and networks
Action Plan for Circular Economy	2024	Climate + Energy	Urban and environmental plans and regulations
Milano Futura Ora Program	2024	Mobility	Programs and programming agreements
Moves	2024	Mobility	Participatory processes and networks
Air and Climate Alliance	2025	Climate + Energy	Participatory processes and networks
Plan to tackle energy poverty and energy precarity (in Approval)	2025	Climate + Energy	Urban and environmental plans and regulations
Green and Landscape Plan (in Elab.)	2025	Green + Biodiv.	Urban and environmental plans and regulations
Strategic and Operational Sustainability Plan for Tourism and Events (in Elab.)	2025	Food + Tourism	Urban and environmental plans and regulations

The aim of this phase was to reconstruct the governance ecology in which sustainability-related policy instruments and governance devices emerge, and to identify the ways in which environmental priorities are translated into programmes, targets, and partnerships. The initial mapping also highlighted the emergence of significant turning points that shaped Milan’s urban environmental trajectory, such as the catalytic mega-events of Expo 2015 and the Milano-Cortina 2026 Winter Olympics (see Section 4).

The sustainability-related policy instruments present in the initial mapping span across multiple domains, including food policy (e.g., Food Policy Milano and related international frameworks), spatial planning and urban regeneration (e.g., railway yards agreements, metropolitan and municipal plans, and international regeneration initiatives), green and biodiversity governance (e.g., regulations on public green spaces and emerging biodiversity strategies), sustainable mobility (e.g., PUMS and traffic management plans), air, energy and climate governance (e.g., PAES, PAC, regulatory and participatory climate devices), and event- and tourism-related sustainability frameworks linked to the Milano-Cortina Winter Olympics.

The initial mapping was followed by an in-depth discourse analysis. Building on the identified sustainability-related policy instruments, we selected a smaller corpus of 11 documents for detailed analysis (see Table 2). The documents were selected according to the following criteria:

1. Their temporal relevance across the identified three major turning points (i.e., urban mega-events and emergencies);
2. Different levels of implementation, including policy instruments that have been adopted, updated versions and instruments that are under elaboration;
3. A high degree of heterogeneity characterizing both the types of policy instruments included (e.g., policies, plans, reports, and presentations) and the actors involved in their development and implementation (e.g., public authorities, private firms, foundations, and civic networks);
4. Their coherence with urban mitigation and adaptation goals (Seawright and Gerring, 2008).

**Table 2:** Selection of analysed sustainability-related policy instruments.

Sustainability-related policy instruments	
(Post) Expo 2015	2015 - Milan’s Food Policy - Policy Guidelines 2016 - The Expo we learned. The Legacy of a Major Event from the Perspective of the Circular Economy
Territorial Government Plan - PGT	2019 - Territorial Government Plan (PGT) - General Report
Air and Climate Plan - PAC	2022 - Air and Climate Plan (PAC) 2022 - Air and Climate Plan (PAC) - Presentation 2023 - Climate City Contract Action Plan 2024 - Climate City Contract - Presentation 2024 - Climate City Contract - Presentation extended version
Milano-Cortina Winter Olympic Games 2026	2019 - Winter Olympics 2026 - Bid Dossier 2023 - Sustainability, Impact and Legacy Report
Sustainable Tourism	2025 - Sustainable Tourism Plan - Presentation

In addition to the documentary corpus, the analysis draws on a complementary set of 10 semi-structured key informant interviews, conducted between December 2024 and September 2025. Interviews were designed to support and enrich the document-based analysis by connecting policy discourse to actor perspectives and coalitional dynamics not fully captured by documentary sources alone. Interviewees were selected based on their institutional role in managing urban environmental issues or their relationship with mega-event governance, particularly the Olympics-related governance network. Four categories of informants were identified *a priori*: public officials and technicians from the municipal administration, environmental consultants, climate policy experts, and private actors involved in initiatives related to the Milano-Cortina 2026 Winter Olympics (see Table 3).

**Table 3:** List of interviewees.

Q. of interviewees	Code	Field of interest
3	(E)	Experts in urban planning, urban policies, urban elites, and urban mega-events
3	(C)	City/urban environmental consultants
2	(PA)	Public officials, political representatives and technicians from the municipal administration
2	(P)	Private actors involved in initiatives related to the Milano-Cortina 2026 Winter Olympics

The interview guide was semi-structured and organised into four to five thematic blocks, the composition of which varied according to the interviewee’s institutional position. Each interview approximately lasted 1 hour and typically opened with a brief self-presentation, in which informants described their professional role and institutional affiliation, followed by a block of questions on urban governance, addressing actor networks, coalition building, decision-making spaces, and dynamics of alliance or conflict. The final block(s) focused more specifically on environmental governance, the management of urban green space, environmental policy, and the institutionalisation of environmental discourse, with particular attention to how these dimensions relate with the urban transformation agenda and the governance of urban mega-events in Milan. The analytical categories used to identify storylines and discourse coalitions (presented in Section 4.3, “Framing Sustainability and Urban Tensions”) were developed inductively through an iterative analysis of the interviews, subsequently refined and substantiated through the analysis of the documentary corpus and literature.

Hence interviews are treated as a supportive and triangulating source rather than a stand-alone dataset analysed independently of the documentary evidence. The interviews allowed us to engage with discourse-analytic insights on storylines, actors and discourse coalitions, dynamics of collaboration and conflict, and decision-making configurations — all particularly relevant where authority is dispersed and policy direction is stabilised through narrative alignment across heterogeneous actors (Hajer, 1997, 2003).

All interviewees received a written information sheet, signed an informed consent form before the interview, authorised audio recording, and were informed of their right to withdraw or request the modification or deletion of their statements. Interview data were anonymised and processed in accordance with Regulation (EU) 2016/679 (GDPR). While full anonymity could not be guaranteed, three measures were applied to protect interviewees. First, interviewees are referred to only by broad institutional category rather than by name, title, or specific organisation (e.g., “a municipal official,” “a private sector representative”). Second, anonymised codes were used to distinguish individual respondents within each category (e.g., PA1, PA2, for public officials; see Table 3). Third, potentially identifying details mentioned during the interviews, were generalised or removed from quoted material. In addition, all research data were securely stored on university-managed local storage systems, where regular back ups are ensured and access is restricted to only authorized members of the research team.

Given the limited number of interviews and the purposive nature of the sampling strategy. Findings derived from interview material are therefore interpreted as indicative rather than generalisable, and are read in conjunction with the broader documentary evidence to mitigate the risk of selection bias inherent in a purposively recruited, key-informant sample.

To deepen the analysis on Milan’s environmental sustainability governance system, the research focused on the identification and analysis of discursive frames found in environmental-related policy instruments (Seawright and Gerring, 2008; Patton, 2002). The coding grid used to identify and classify these frames was developed inductively through an iterative process (Gioia et al., 2013). First, we analysed the semi-structured interviews to develop an initial set of interpretive hypotheses concerning the discursive frames underpinning urban sustainability-related policies and to generate a preliminary set of keywords associated with each frame. Subsequently, we conducted a literature review and began the document analysis to refine the substantive content and semantic boundaries of the emerging frames. This engagement with documentary and theoretical sources enabled progressive refinement of the coding grid across coding cycles, in which codes were added, merged, clarified, and redefined based on evidence from both the interviews and the policy instruments.

The selected corpus of 11 environmental-related policy instruments was analysed exhaustively in NVivo following a qualitative analytical approach (see Table 4 for the final coding grid). The coding process began with the identification of the term “sustainability” and its stemmed variations across the selected policy instruments. By using the identified keywords and discursive frame’s description only as analytical indicators and after careful consideration of the semantic context of relevant excerpts, these were classified according to the analytical coding grid, with codes progressively refined through two individual coding cycles. To enhance reliability, cross-coding review was conducted collaboratively among two research team members. Disagreements in code assignment were resolved through discussion and consensus-building, ensuring consistency in the application of the coding grid across the corpus. Through NVivo’s analytical functions, the percentage distribution of citations within each discourse framework (code) was calculated for each analysed document, generating quantitative data that enabled comparative visualisation of how sustainability-related frames are weighted and distributed across the policy instruments (see sections 4.3 and 4.4). Rather than adopting theoretical saturation as a stopping criterion, all 11 documents were coded systematically to provide a comprehensive overview of sustainability-related discourse across the selected corpus.

**Table 4:** Code grid. Discursive frames and keywords.

Keywords*	Description	Discursive frames
right, commos, inclusion, participation, justice, equity, climate justice, inclusivity, empowerment, equo, intersectional, ethical, care, common-pool.	Interprets environmental sustainability as a right and a common good, with an emphasis on inclusion, justice, equity, and civic participation (Whitehead, 2013; Foster and Iaione, 2015)	F1. Commons & Justice
complexity, metabolism, resilience, adaptation, nature-based solutions (NBS), ecosystem, biodiversity, landscape, protection, restoration, socio-ecological system, biosphere, interconnection, transformability, dynamic systems.	Urban environmental sustainability as a complex system. Represents the city to be made resilient and adaptive through integrated approaches and nature-based solutions (Nelson et al., 2007; Folke, 2016)	F2. Systemic-Metabolic
targets, KPIs, accountability, neutrality 2030/2050, capacity-building, efficiency, quantification, criteria, innovation, practices, construction, infrastructure, protocols, standards, objectives, data, data-driven, big data, smart, productivity, open data, competitiveness, impact.	Reduces environmental sustainability issues to numerical targets, performance indicators, and accountability tools (Bibri and Krogstie, 2020; Kitchin, 2014)	F3. Technocratic-quantificationist
branding, attractiveness, smart city, green city, reputation, market, flagship, investors, PPPs, legacy, coalition, certifications, competitiveness, labels, credentials, eco-city, low-carbon city, zero-net-energy, eco-gentrification, green urban renewal, urban branding, place marketing.	Uses environmental sustainability as a resource to enhance urban competitiveness, investor attractiveness, and international reputation (Harvey, 1989; Cucca, 2012)	F4. Asset-Branding

*Keywords were used as analytical indicators rather than direct proxies for interpretative frames. Their classification was based on the semantic context in which they appeared during document analysis.

The resulting typological distinction enables a comprehensive discussion on environmental-related policy instruments in Milan and a detailed analysis of the various ways in which these are formulated and implemented. While centred on a limited number of policy instruments, the analysis provides a meaningful overview of the mechanisms of sense-making embedded within UEG devices.

## 4 Findings

### 4.1 Turning points in Milan’s socio-ecological governance (2015–2025)

The evolution of UEG in Milan between 2015 and 2025 can be interpreted through a sequence of conjunctural turning points that repeatedly reconfigure policy priorities, actor coalitions, governance devices. These turning points do not constitute stages in a cumulative transition trajectory but instead operate as policy windows in which environmental sustainability is selectively redefined, new governance devices are introduced, and existing instruments are recombined without producing long-term strategic synthesis. Across the period analysed, the governance of environmental sustainability takes shape through successive waves of policy activation, with the result of a layered governance configuration in which instruments accumulate over time, coordination remains partial and coherence is episodic.

1. the *post-Expo moment* (2015–2018), during which sustainability is increasingly articulated in relation to international initiatives and visibility-oriented discourses;
2. the *climate emergency declaration and the COVID-19 period* (2019–2021), which accelerates adaptation- and health-related rationales and consolidates new participatory interventions;
3. the *preparation of the Milano-Cortina Winter Olympic*s (2022–2025/6), which expands the repertoire of instruments and partnerships through “legacy-oriented” policy languages and event-oriented sustainability standards.

**Figure 1:**
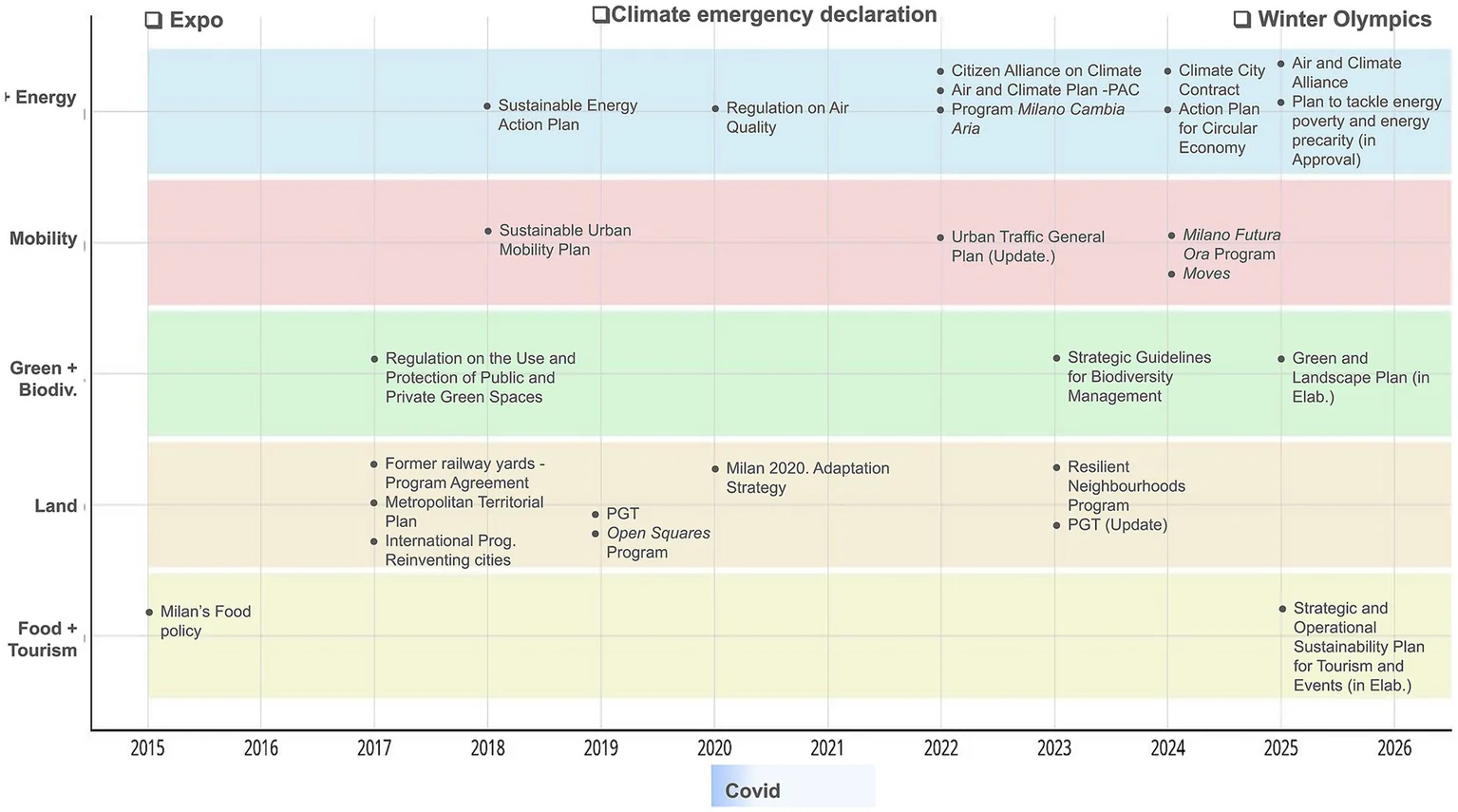
Distribution of sustainability-related policy instruments across turning points. Authors’ elaboration.

Figure 1 provides a synthetic overview of this process, visualising the temporal distribution and accumulation of relevant sustainability-related policy instruments, including the corpus of 11 cases further deepened in this study, across the identified turning points.

Figure 1 clarifies not only the multiplication of instruments, but also their sectoral distribution and temporal clustering around turning points:

*Around Expo 2015: Sustainability and green reputation*: This turning point is characterised by the consolidation of policy instruments that connect sustainability to food systems, green reputation, and selective urban regeneration. The environment acts as a reputational lever and as a narrative for global positioning. After Expo 2015, whose theme *Feeding the planet, energy for life,* guided the adoption of *Milan’s Food Policy*, new tools and projects aimed at fostering sustainable practices in food production and consumption were consolidated, often through public-private partnerships and local initiatives such as the *Hub di Quartiere* (Neighborhood Hubs), designed to counter food waste and support people in difficulty. From an urban planning perspective, urban environmental policies have become increasingly territorial, as reflected in measures such as the 2017 *Regulation on Public and Green Spaces,* and the adoption of Milan’s main planning instrument, the *Territorial Government Plan (PGT)* which prioritizes urban regeneration alongside sustainability and resilience. Consequently, a series of strategic urban initiatives oriented toward redevelopment and regeneration were launched, such as the *Program Agreement for the regeneration of the former railway yards*, and the participation in the first edition of C40 *Reinventing Cities*, an international program that incorporates, in an unprecedented way, environmental discourse and the dimension of sustainability. These initiatives helped define sets of projects and discursive frameworks of reference that would inform the subsequent update of the PGT, and would later appear in many instruments of subsequent phases, such as the *Climate City Contract* and the *Milan-Cortina 2026 Winter Olympics Bid*.

The Expo 2015 opened an exceptional opportunity for the affirmation of environmental sustainability as a key element of a reputational strategy, enabling the formation of new public-private coalitions that brought together national and international real estate developers (such as Pirelli RE and Hines), newly established entities (like Fondazione Fiera), and public actors including the Municipality, the Region, and the Metropolitan City. In the following years, the Expo experience also contributed to the expansion of network-based partnerships involving universities (such as the Milan Polytechnic University), foundations, technical agencies (such as the Italian State Railways), international city networks (such as “100 Resilient Cities”), and a wide range of private actors, particularly around strategic regeneration initiatives and innovation districts.

*Emergencies: between environment and health*: The emergency phase introduces a turning point, where climate risk, air quality, and public health rationales become more prominent and are associated with spatial and mobility interventions. The declaration of a climate emergency and the COVID-19 public health emergency open a window of opportunity that accelerates the adoption of measures related to air quality, adaptation, and land use, and promotes discursive institutionalization. The *Milan 2020. Adaptation Strategy*, for example, engages with policies already underway, such as *Open Squares*, and envisages widespread actions to improve public space, increase urban vegetation through new green designs, and promote slow mobility.

In this context, the lexicon of emergency also reshapes hierarchies of legitimacy among urban problems. Air quality, heat exposure, health vulnerability, and the scarcity of accessible open spaces become more central not only because they are objectified through epidemiological and climatic data, but also because they can be translated into administratively manageable categories capable of justifying rapid and visible interventions. In this sense, public health operates as a bridging frame, making environmental issues more immediately legible and politically mobilizable while linking ecological concerns with everyday well-being.

At the same time, this convergence tends to privilege measures compatible with already established techno-spatial repertoires—such as pedestrianisation, urban cooling strategies, urban forestry, traffic calming, and tactical urbanism—without necessarily opening a broader discussion on the structural determinants of socio-ecological vulnerability, including the unequal distribution of urban green space, housing conditions, real estate pressures, or the differential exposure to pollution across neighborhoods and social groups. The result is a configuration in which emergency does not merely accelerate public action but also selectively prioritizes particular policy responses. Interventions that can be framed as simultaneously environmental, health-oriented, and readily implementable gain greater prominence, while measures requiring redistribution, stronger regulation of urban valorization processes, or a reconsideration of prevailing growth models remain weaker or intermittent.

*Olympic environment: legacy and sustainability*: As Milan-Cortina 2026 Winter Olympics approach, new alliances and narratives are activated, oriented toward sustainability as an infrastructural and reputational legacy. Sustainability narratives increasingly operate through event-oriented frameworks and the proliferation of instruments on climate and air quality and mobility, alongside formalised participation formats and event-related environmental standards often centered on the involvement of private actors. A key example is the *Air and Climate Plan (PAC)*, which represents a cornerstone in the commitment of the city to mitigation and adaptation to climate change and carbon neutrality by 2050. The PAC defines a set of goals, priorities and strategic actions, while integrating international principles and networks with structured forms of participation including citizens, economic actors, the third sector, and academia.

On the other hand, in the land domain, there is a shift from the concept of “regeneration” to “resilience”; for example, the *Resilient Neighbourhoods Program* operates at the local scale through integrated actions, in synergy with the *PAC* and *Open Squares*, with the aim of maximizing climate-adaptation effects also at the neighborhood level. Additionally, in this regard, the city of Milan is going through a particularly demanding phase, dictated by the update of the *PGT*, the delivery of the projects framed within the *Italian National Recovery and Resilience Plan (PNRR)*, and the kick-off of large strategic urban regeneration projects in connection with the Milan-Cortina 2026 Winter Olympics.

Framed within the actions encompassed by the PAC, and fostered by international initiatives such as the European mission of *100 Climate-neutral and Smart Cities*, new coalitions in the form of formalized agreements aimed at informing the process of climate governance have also been adopted. That is the case of the *Climate City Contract,* an international agreement that brings together a myriad of actors through which Milan commits to achieving climate neutrality by 2030. In the case of Milan, the *Climate City Contract* introduces an elitist-type coalition. The modes of involvement adopted have been selective and top-down, in contrast with the open calls through which most other municipalities in the European network have chosen to proceed. The Municipality of Milan instead has privileged a strategic and relational approach, extending a direct invitation to their publicly owned companies and operators (ATM, A2A, MM), public and private universities, private foundations (Cariplo, FAI), housing cooperatives, environmental associations (Legambiente), and major real estate developers and urban regeneration actors (Coima, Covivio, LandLease, REDO). An experience that, alongside other recently adopted participatory devices aimed at formalizing the commitment of private firms to the priorities and set of actions defined within the PAC (such as the *Air and Climate Alliance*), reinforces the tendency toward selective public–private coalition building, often privileging the presence of influential private actors.

Similarly, as the Milan-Cortina 2026 Winter Olympics approach, the management of the “sustainable Olympics” is witnessing the formation of constellations of highly institutionalised and capitalised actors often converging locally within new operational bodies such as Fondazione Milano Cortina. For instance, on one hand there are international bodies (IOC, IPC), and national and local public institutions and bodies (CONI, regional and municipal administrations, Infrastructure Society Milano-Cortina 2026, AMAT); while on the other, private actors such as premium and official sponsors, and a myriad of firms involved in service provision, commerce and acquisitions. Among the latter, particularly relevant is the central role of global investors and real estate developers in processes of green urban regeneration and infrastructures for urban mega-events (Coima, Covivio, Eventim, Lendlease, Nhood, REDO). This concentration of highly capitalised, real estate-oriented actors around the Olympic effort foregrounds a distributive question central to debates on urban growth machines and urban justice (Logan and Molotch, 1987; Fainstein, 2010), one that has been examined with specific reference to Milan’s financialised growth coalition (Vicari and Anselmi, 2020).

In this scenario, two instruments currently under development also emerge as particularly relevant: the *Strategic and Operational Sustainability Plan for Tourism and Events*, and the *Green and Landscape Plan*. Both aim to define an infrastructural and reputational legacy consistent with Milan’s growing positioning as a “green” city and a sustainable tourist destination. Moreover, as of December 2025, the *Minimum Environmental Criteria (CAM)*, required for events organized or supported by the municipal administration have entered into force.

### 4.2 Sustainability molecules and urban environmental governance

These turning points show how Milan’s socio-ecological governance evolves through successive waves of policy activation and instrument proliferation, without converging into a single strategic architecture. Over time, this process has produced a dense and heterogeneous governance landscape in which socio-ecological change is unevenly distributed across policy domains, territories, and social groups. Milan’s experience suggests that turning points function not only as moments of opportunity, but also as mechanisms of selection. They privilege forms of environmental action that are compatible with reputational strategies, metric-based governance, and event-driven development, while marginalising claims and practices that challenge dominant urban growth trajectories.

The proliferation of sustainability-related policy instruments observed between 2015 and 2025 does not amount to a fragmented or incomplete transition in a conventional sense. It reveals instead a specific configuration of policy instruments through which socio-ecological change is governed and sustainability is rendered actionable. These partially autonomous instruments coexist, interact, and occasionally align without producing a unified strategic synthesis. Within this configuration, individual policy instruments operate *as the nuclei of sustainability molecules, binding to themselves the actors, frames, indicators, and resources through which they organise environmental action*, across domains such as mobility, air quality, food systems, tourism, and neighbourhood regeneration. Plans, programmes, regulatory tools, participatory formats, and event-driven strategies operate as relatively self-contained governance devices, while remaining relationally entangled through shared narratives, overlapping actor constellations, and common repertoires of indicators and performance metrics. *Sustainability molecules* should be understood not only as discursive and institutional configurations but also as enabling or constraining specific bundles of social practices (Reckwitz, 2002; Shove et al., 2012).

This *molecular* structure helps explain why coordination in Milan’s environmental governance recurs but seldom endures. Alignment does occur, especially at conjunctural turning points when political attention, financial resources, and symbolic capital coincide. However, this alignment typically remains a short-lived recomposition, not a stable form of integration. Over time, new instruments and initiatives are introduced without displacing existing ones. The outcome is institutional layering, not consolidation. Coherence tends to surface in discrete episodes, often around flagship programmes or forward-looking horizons, and then weakens as priorities change and additional devices enter the governance landscape.

*Molecularisation* should therefore not be understood merely as an organisational feature of governance arrangements, nor as a property of governance itself. Rather, it refers to a specific mode of governing, in which transition is decomposed into partially autonomous yet relationally connected *molecules*. These *molecules* differ in their actors contellations, evidentiary regimes, temporal horizons, and capacity to connect with other instruments; their coordination is therefore episodic, emerging around policy windows, funding opportunities, crises, or mega-events, but rarely consolidates into a stable strategic architecture.

A particularly illustrative example of this molecularised policy configuration is provided by the *Climate City Contract*. Conceived as an integrative and mission-oriented instrument, the *Contract* articulates sustainability through systemic language, collective responsibility, and multi-actor engagement. In practice, however, its operationalisation relies heavily on targets, milestones, and reporting requirements aligned with European governance frameworks. This duality allows the *Contract* to function as a coordination device across heterogeneous policy domains, while simultaneously narrowing the space for political contestation by translating complex socio-ecological questions into deliverable-oriented commitments. The *Climate City Contract* thus exemplifies how molecularised policy instruments can enable alignment while reshaping the meaning of participation and transformation (see Section 4.3).

These dynamics suggest that a governance field, structured by *molecularised* policy instruments, is both enabling and constraining. It facilitates action in complex institutional environments by decomposing sustainability into governable units, yet it also introduces a selective closure of the field of political possibility. Claims, temporalities, and practices that cannot be translated into discrete instruments, indicators, or performance frameworks struggle to gain traction. Conflict is not eliminated, but it is channelled into technical and procedural domains, reducing the likelihood of challenges to dominant urban growth trajectories.

In this sense, the *molecularisation* of policy instruments within UEG helps explain why socio-ecological change in Milan advances unevenly across policy domains, territories, and social groups. Sustainability becomes actionable through discrete instruments, indicators, partnerships, and temporal horizons, but this actionability does not automatically produce systemic transformation.

### 4.3 Framing sustainability and urban tensions

As anticipated in the methodological section, discursive frames were identified through a combined inductive–deductive strategy. Preliminary discursive frames were initially identified from the interview material, allowing recurrent themes and interpretative categories to emerge. They were iteratively refined through close reading of the selected policy documents and triangulated with the international literature on urban sustainability and climate governance, which recurrently highlights tensions between justice-oriented and market-oriented visions, as well as between systemic and technocratic approaches. This process supported the identification of recurring semantic clusters, narrative oppositions, and framing patterns (see Table 4).

**Figure 2:**
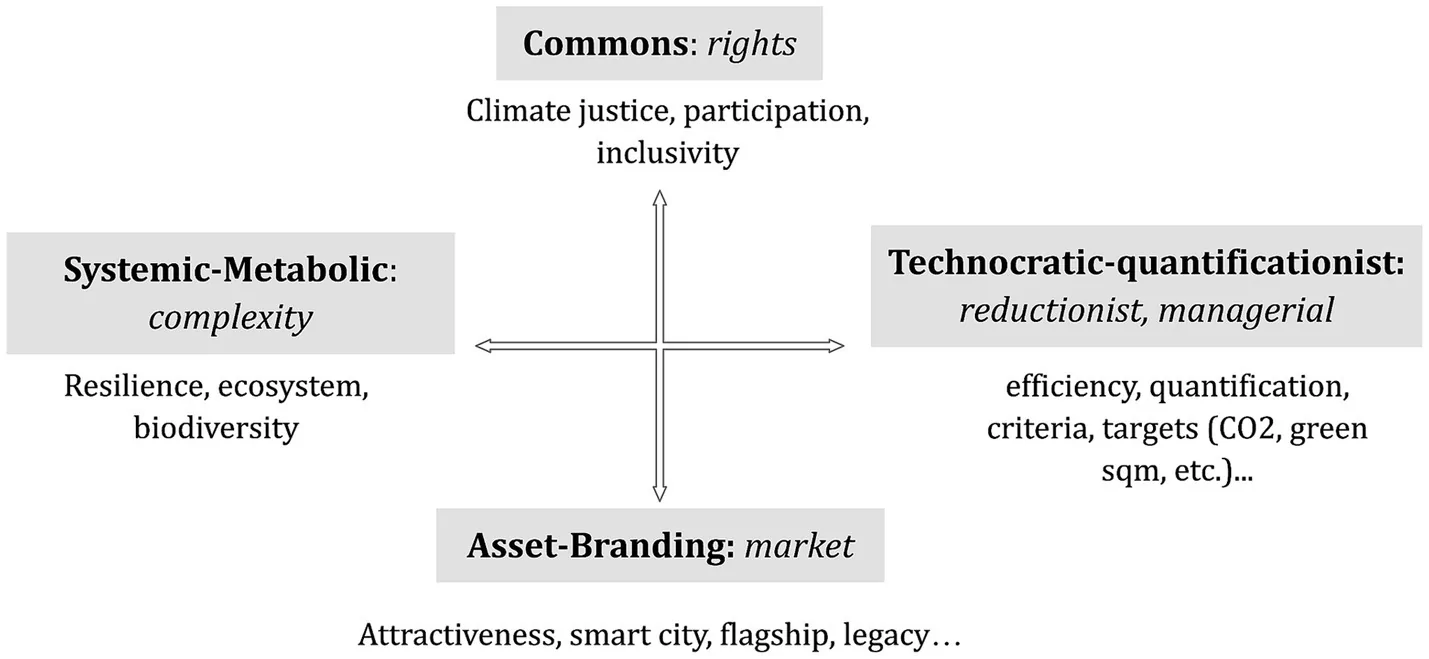
Intersection of cross-cutting interpretative axes. Authors’ elaboration.

Through this process, two cross-cutting interpretative axes emerged as particularly salient in structuring Milan’s discursive field of sustainability, capturing the underlying tensions that run across documents, instruments, and governance devices, and shaping how sustainability is problematised and operationalised across *sustainability molecules*. Figure 2 schematically represents these axes and their intersection. These axes correspond to the four interpretative frames introduced in the coding grid, which organise the discursive field along two underlying tensions: *justice* versus *asset-based* interpretations of sustainability, and *systemic* versus *technocratic* modes of operationalisation. The axes should therefore be understood as analytical tools that make visible the relational positioning of *sustainability molecules* within a fragmented governance field, and not as a normative benchmark.

The vertical axis contrasts two fundamentally different interpretations of the meaning and purpose of sustainability. At one end, sustainability is framed as a collective good and an urban right, closely associated with environmental justice and democratic claims over how cities are shaped (e.i. redistribution, participation, inclusivity, etc.). In this perspective, sustainability is linked to the right to the city claims, to fair access to environmental resources and protections, and to efforts to reduce socio-spatial inequalities. Policies articulated within this frame tend to emphasise the unequal distribution of environmental harms and benefits across urban populations.

At the opposite end, sustainability is framed as an asset, as a strategic resource for enhancing urban competitiveness, strengthening international reputation, attracting investment, talent, and visitors. Here, sustainability operates as part of broader place-branding strategies and market-oriented development narratives, where environmental commitments are mobilised to position the city within global networks, rankings, and flagship initiatives, often prioritising visibility, scalability, attractiveness and symbolic value. The asset-branding frame can also be interpreted through Reckwitz’s (2020) notion of urban singularisation, whereby sustainability becomes a symbolic resource through which cities seek to position themselves as unique, innovative, globally attractive.

For instance, in Milan, particularly in the period following Expo 2015, the concepts of sustainability and urban resilience have been progressively incorporated into political and administrative discourse, undergoing significant processes of reinterpretation and institutionalisation. As noted by one public administration representative:

*“Initially, it was not at all clear what it actually meant to deal with resilience at the urban scale […] there were multiple and different ways of understanding the resilience paradigm as applied to the city […] Over time, there was an effort to develop a specific way of interpreting resilience, which, quite simply, means deciding what to prioritise, in a sense. That is, deciding how the city administration chooses to use the framework, the direction, or the resources it has gradually gained access to, in order to try to respond to what it believes are the main risks facing the city itself” (PA2).*

However, alongside a growing recognition of environmentally oriented frames, Milan has also increasingly become a terrain in which sustainability is framed as a competitive economic asset. This framing has been reinforced by logics of urban entrepreneurship, international positioning, and city attractiveness, which tend to privilege visibility, investment appeal, and reputational gains. As a result, sustainability is often mobilised through urban marketing strategies and flagship projects, frequently driven by real estate profitability and weakly connected to more comprehensive, long-term approaches of urban sustainability planning.

*“There is close attention to individual buildings, yet overall there is no comprehensive environmental assessment. Such an evaluation has either not been carried out, or, if it is underway, it is not publicly accessible” (E2).*

*“Too often, investments are channelled into highly selective interventions: the large construction site for a flagship park that becomes a symbolic project, rather than a more even, city-wide distribution of green spaces […] While this may be less effective from a marketing perspective or in terms of ribbon-cutting visibility, it delivers far greater benefits for public health and for the city as a whole. This calls for fewer flagship interventions and a more widespread, capillary allocation of resources across the urban territory” (E3).*

This vertical axis captures a central tension in Milan’s trajectory, where sustainability can be positioned as a constraint on market logic or, conversely, as a lever for market expansion and international attractiveness. The analysis indicates that both orientations circulate across the policy landscape and sometimes appear within the very same instruments. This overlapping tends to produce ambivalence, more than a clear and consistent strategic direction.

The horizontal axis concerns how sustainability is methodologically and operationally understood. At one end, a systemic-metabolic approach frames the city as a complex socio-ecological system characterised by interdependencies, long-term processes, and cumulative transformations. Sustainability, in this view, requires integrated approaches that address urban metabolism, biodiversity, ecosystem services, and social practices alongside material infrastructures.

At the opposite end, a technocratic-quantificationist approach translates sustainability into measurable targets, indicators, benchmarks, performance metrics. In this framing, complexity becomes “governable” because it is translated into numbers, made comparable and controllable through quantification and standardisation, addressed with linear problem-solving logics. Hence, the emphasis shifts to efficiency, monitoring, reporting, and compliance, often prioritizing what can be counted (such as emissions reductions, square metres of green space, numbers of trees planted, etc.), over qualitative or relational dimensions.

In Milan, this polarization in how sustainability is operationalised opens space for positivist and reductionist approaches that tend to translate ecosystemic complexity into quantifiable indicators, often stripping sustainability of its political and relational dimensions. This logic is evident, for instance, in the language of “legacy and impact” that shapes narratives around urban mega-events such as the Milan-Cortina 2026 Winter Olympics.

*“With regard to all the environmental aspects, the main objective is to reduce CO₂ emissions related to the staging of the event [Milan-Cortina 2026 Winter Olympics] compared to previous editions, and to address everything that remains outside that scope, thereby to minimise emissions as much as possible and then offset what are considered standard or unavoidable emissions” (P2).*

*“Obviously yes, the Olympic and Paralympic Village will be equipped with all the necessary tools to achieve, in that sense, a zero impact. That is, with the Olympic Village there should be no energy consumption, thanks to renewable energy sources” (PA1).*

A similar dynamic can be observed in the emergence and widespread adoption of concepts such as nature-based solutions, which are frequently framed through the quantification of green indicators (such as emissions reductions or the adoption of smart technologies) while systematically neglecting qualitative dimensions that account for an ecosystemic understanding of urban sustainability.

*“Everyone talks about nature-based solutions, but often inappropriately. A balcony with a tree is not a nature-based solution […] A park that is extremely well designed but built over underground parking and other infrastructure is not fully permeable land […] It may even be a very unsustainable park in terms of the plant species selected and their composition […] trees are not numbers […] We should not simply count how many trees we plant; we need to consider which species they are, how they are planted, and what surrounds and lies beneath them. Otherwise, we keep making the same mistake of reducing everything to numbers […] what is never counted is how many of these trees are under stress, how many die in the first year, and how much support they require and at what cost. Not economic cost, but in terms of water, which is a precious resource and one we are increasingly becoming aware of, along with everything else involved” (C1).*

This horizontal axis reveals a tension between sustainability as an open-ended process of systemic transformations and sustainability as a managerial problem to be optimised; as documented in the analysis, Milan’s policy instruments frequently oscillate between these two orientations, producing hybrid configurations that combine systemic language with technocratic practices.

The intersection of these two axes generates *four dominant discursive frames*, corresponding to the four quadrants represented in Figure 2. These discursive frames are not ideal types imposed ex ante, they crystallise from the ways in which *sustainability molecules* combine orientations along both axes.

1. First, the intersection of commons/justice and systemic-metabolic orientations produces a frame of *sustainability as eco-social justice*, where sustainability is conceived as a redistributive and transformative project, rooted in local territories and attentive to social and ecological vulnerabilities. Participation, co-design, collaboration, inclusion, and the right to the city are central, and urban nature is framed as a collective infrastructure of care and wellbeing.
2. Second, the combination of commons/justice orientation with a technocratic-administrative produces a frame of *sustainability as a technically administered common good*. Equity and inclusion remain normative references, but they are translated into formalised procedures, codes, and regulatory instruments; participation is institutionalised and proceduralised, prioritising manageability and administrative coherence over political contestation.
3. Third, the intersection of asset-branding and systemic-metabolic orientations generates a frame of *sustainability as a competitive asset of the global city* where sustainability is articulated through holistic and future-oriented language (resilience, integration, climate neutrality…) while being mobilised to enhance international visibility and reputational capital. Mega-events, flagship initiatives, global networks play a central role in this configuration.
4. Finally, the combination of asset-branding with technocratic-quantificationist approach produces a frame of *sustainability as technical-quantitative performance.* This frame is particularly prominent in strategic documents, where sustainability is defined through KPIs, emission targets, timelines, and deliverables; and climate action is framed as a management challenge, prioritising efficiency, accountability, and measurable outputs.

**Figure 3:**
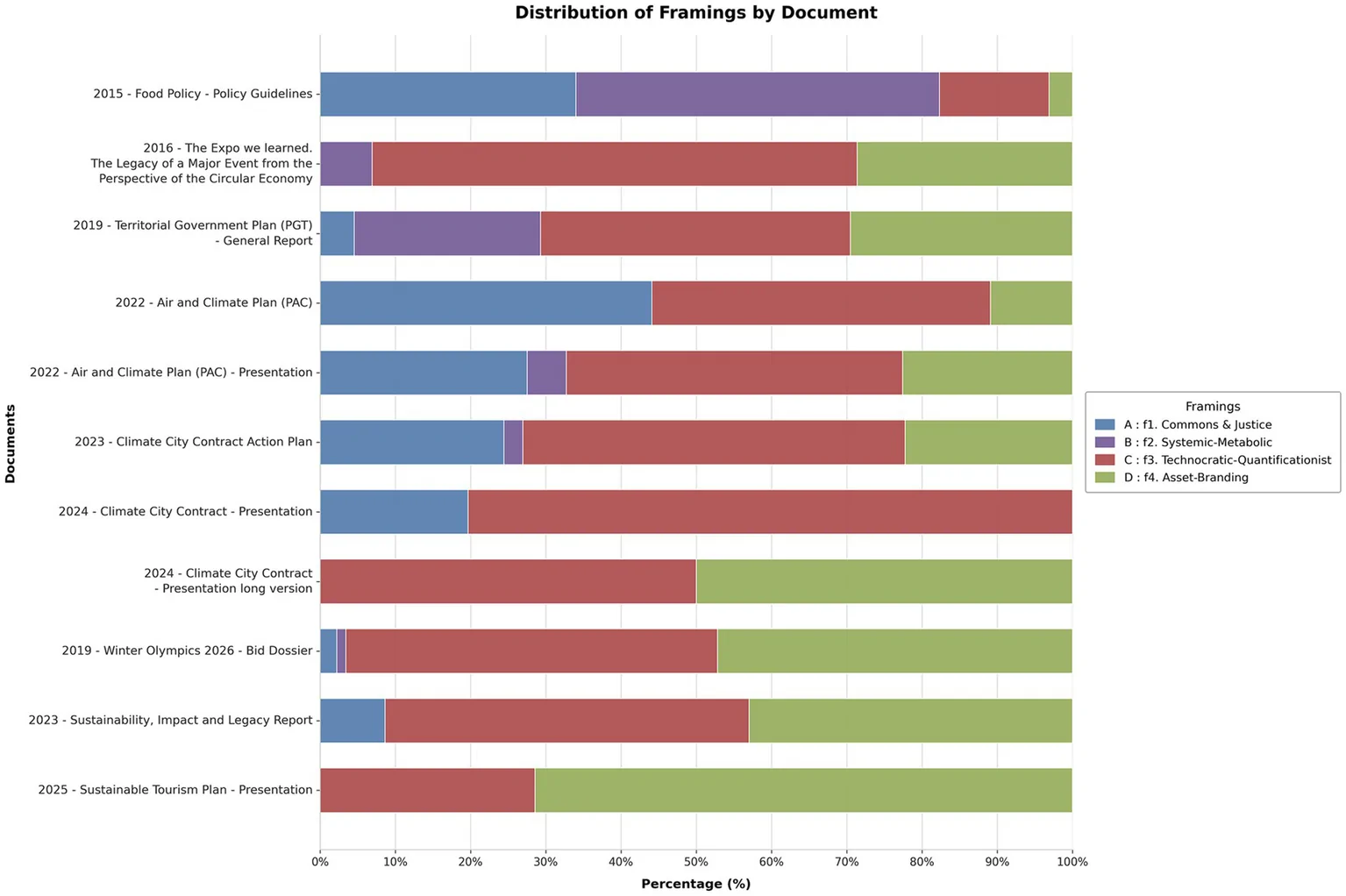
Discursive frame distribution of analysed sustainability-related policy instruments. Authors’ elaboration using NVivo’s analytical functions and Microsoft Excel, based on the data resulted from calculating the percentage distribution of citations within each discourse framework of the corpus of 11 analysed environmental-related policy instruments.

Considering the 11 relevant sustainability related policy instruments analysed in depth (see Section 3), Figure 3 visualises how discourses on sustainability are distributed across frames. Most instruments display hybrid configurations, combining elements of different discursive frames. Overarching plans such as the *Air and Climate Plan* or participatory instruments such as the *Climate City Contract* tend to mobilise systemic and justice-oriented language while simultaneously relying on technocratic indicators and performance targets. Event-related documents linked to Expo 2015 and Milano-Cortina 2026 Winter Olympics show a stronger concentration along the asset-branding axis, often coupled with technocratic quantificationism/reductionism.

Such a distribution confirms that sustainability in Milan operates as a plural and contested discursive field, in which environmental justice, global attractiveness, technical governance, and strategic urban imaginaries coexist and compete. The frames function as lenses through which actors interpret the climate transition, select priorities, form coalitions and define what counts as legitimate action. In doing so, they produce concrete governance effects, shaping urban form, investment priorities, access to resources, and the distribution of environmental benefits and risks.

The analysis suggests that environmental sustainability in Milan does not function as a stable unified paradigm but it unfolds as a dynamic and contested arena in which meaning, priorities and coalition are periodically reconfigured in relation to turning points and policy windows. *Sustainability molecules* constitute the material and discursive infrastructure through which this process is governed, making visible the tension between transformation and legitimation that characterise Milan’s urban environmental transition.

### 4.4 From discursive frames to selective governance effects

Discursive frames do not simply describe different meanings of sustainability, they shape the unequal institutional durability of *sustainability molecules*; they define which claims become administratively manageable, which actors are recognised as legimimate partners, and which forms of environmental action can be stabilised across policy domains.

The analysis of the 11 sustainability-related policy instruments considered in depth shows that *sustainability molecules* positioned along the asset-oriented and technocratic ends of the two interpretative axes tend to acquire greater institutional stability, because they are more easily aligned with performance metrics, reporting requirements, funding mechanism and international policy circuits. As a result, they are more likely to function as coordination hubs within the broader governance landscape, particularly during conjunctural phases associated with mega-events and anticipatory planning.

By contrast, *sustainability molecules* grounded in commons- and justice-oriented framings, especially when coupled with systemic–metabolic perspectives, display a more fragile institutional anchoring. While these orientations are recurrent in participatory processes, neighbourhood-scale initiatives, and biodiversity-related policies, their capacity to shape the overall direction of UEG remains limited. Such molecules often depend on temporary political support, project-based funding, or individual champions, and struggle to be stabilised through the same metric-based and procedural mechanisms that sustain more technocratic configurations.

**Figure 4:**
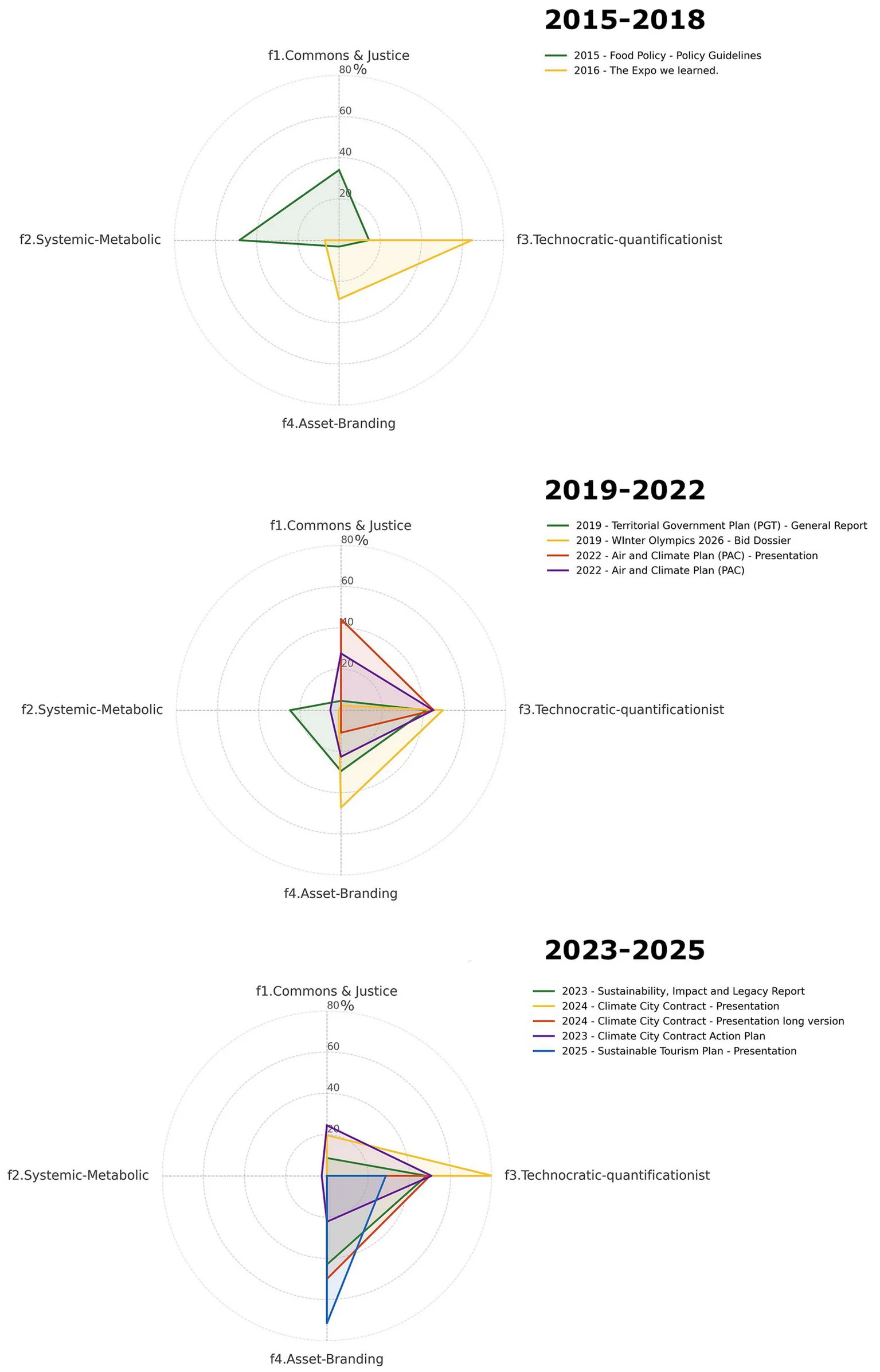
Temporal and discursive frame distribution of analysed sustainability-related policy instruments. Authors’ elaboration using NVivo’s analytical functions and Microsoft Excel, based on the data resulted from calculating the percentage distribution of citations within each discourse framework of the corpus of 11 analysed environmental-related policy instruments.

This pattern reinforces when adopting a temporal perspective. When observing how our selection of analysed environmental policy-related instruments distributes across the interpretative frames, this resulted in a progressive shift towards a discursive frame of sustainability as *technical-quantitative performance* (see Figure 4). The shift becomes evident in the years around the emergency period, where the systemic-metabolic discourse on sustainability became less present, contrasting with the predominant technocratic-quantitative approach. This transition is rendered even more skewed in the period 2023–2025, with a progressive weakening of the commons and justice discourse, revealing a cluster of instruments, organises around the mega-event of Milano-Cortina 2026 and the *EU Mission on Climate-Neutral and Smart Cities*, that tends to privilege actions centred on management efficiency, accountability and reputation. Thereby, fostering governance restructuring oriented towards the production of and alignment to measurable outputs.

This tendency, however, does not imply that systemic or justice-oriented discourses are absent. Milan’s sustainability governance does not operate through a single dominant framing, but most molecules exhibit hybrid configurations, with overarching plans such as the *Air and Climate Plan* and integrative instruments such as the *Climate City Contract* mobilising systemic and justice-oriented language while simultaneously relying on indicators, targets, and timelines characteristic of technocratic governance. The point, therefore, is not the replacement of justice-oriented or systemic frames by technocratic ones, but their selective translation into formats compatible with administrative routines, reporting obligations, and reputational policy circuits.

While Milan has strengthened its competitive capacity, the private wealth generated by real estate-led development appears disproportionate to the public benefits produced, with redistributive outcomes that remain contested in relation to housing affordability and social inclusion (Bortolotti and Goldstein, 2025; Bricocoli and Peverini, 2024; Wolfgring and Peverini, 2024). Understood relationally, vulnerability is not an attribute of predefined groups but a condition produced through uneven exposure and unequal access (Butler, 2004; Fineman, 2008; Henrique and Tschakert, 2020). This shapes which vulnerabilities are recognised: governance privileges those translatable into targets and measurable interventions (air quality, heat stress, access to green space) while the structural conditions that produce them, such as differential exposure to pollution, housing pressures, and the social costs of urban regeneration, remain less visible and harder to stabilise within the policy agenda.

Across the mapped instruments, participation appears predominantly in institutionally curated forms (consultation, stakeholder engagement, deliberative arenas such as the *Citizens’ Assembly for Climate*, and invited coalition-building) which differ markedly in access, representativeness, and influence. Document analysis and key-informant interviews indicate that the most influential instruments rely on selective, top-down engagement: the *Climate City Contrac*t and the *Air and Climate Alliance*, for instance, build coalitions through direct invitation to public agencies, utilities, universities, foundations, and major private actors (see Section 4.1). Grassroots mobilisation and conflictual forms of participation (Cugnata et al., 2024), by contrast, remain largely external to these formal arrangements (an absence that is itself indicative of how selectively participation is institutionalised within Milan’s sustainability governance).

These patterns reveal a recurrent coalitional logic. Across turning points, the most consequential coalitions form around flagship instruments (post-Expo regeneration, the *Climate City Contract*, the Milan–Cortina 2026 agenda) aligning the municipality, publicly owned utilities (ATM, A2A, MM), universities, bank-origin foundations (Cariplo, FAI), and major real estate developers (Coima, Covivio, Lendlease, REDO), each contributing distinct resources: public authority and land, technical expertise, philanthropic and private capital, and reputational reach. They converge on growth-compatible, metric-legible objectives, while redistributive and grassroots claims remain external, so that coalitions do not merely support but actively orient governance priorities towards asset-oriented and technocratic frames, where actors can most readily stabilise.

## 5 Conclusion: the selective governance of fragmented transitions

This article has argued that Milan’s socio-ecological transition should not be understood primarily as an incomplete movement towards policy coherence, but as a *molecularised* mode of urban environmental governance. The case shows that fragmentation is not only a coordination problem, but also a governing condition through which sustainability is decomposed into discrete policy instruments, made administratively actionable, and selectively aligned with specific coalitions, metrics, temporal horizons, and development priorities.

The article makes three contributions to debates on urban environmental governance and socio-ecological transition. First, it conceptualises *sustainability molecules* as bounded yet relational policy configurations composed of discursive frames, actor constellations, evidentiary regimes, resources, and temporalities. This concept shifts attention from the density or coherence of sustainability policies to the ways in which they connect, stabilise, and acquire unequal coordinating capacity within fragmented governance landscapes. Second, the article shows that *molecularisation* operates simultaneously as a mechanism of coordination and selection. It enables policy activity under conditions of institutional fragmentation, while privileging forms of sustainability compatible with metric-based governance, reputational strategies, public-private coalitions, and event-led development. Third, it advances a sociological reading of urban transition as a field of symbolic struggle and coalition-building, where the legitimacy of environmental action depends on which actors, vulnerabilities, practices, and futures are made institutionally visible.

Seen from this perspective, Milan does not appear as a frontrunner moving along a clearly defined transition pathway, but as a contested governance arena in which socio-ecological change is periodically reassembled through conjunctural alignments. Turning points such as Expo 2015, the declaration of the climate emergency, the COVID-19 pandemic, and the Olympic horizon expand the repertoire of sustainability action, but they also reorganise tensions between transformation and legitimation, systemic change and administrative governability, justice-oriented claims and growth-compatible policy agendas. What emerges, therefore, is not the absence of transformation, but its selective and politically conditioned organisation.

The analysis is limited by its focus on a single critical case and by a research design centred primarily on policy documents and key-informant interviews. The findings are therefore not intended as statistically generalisable, but as analytically transferable to cities shaped by similar combinations of entrepreneurial urbanism, financialised development, and event-led governance. Future research should compare *molecular* configurations across different urban contexts and examine the conditions under which *sustainability molecules* may be redirected towards more redistributive and transformative socio-ecological transitions. Lastly, since our analysis focuses on governance arrangements and policy dispositifs, a further promising future research could also examine how *sustainability molecules* are translated into everyday social practices and whether these reproduce, transform or contest the dominant sustainability frames identified here.

## Data Availability

The study is based on publicly available policy documents and qualitative interview materials. The policy documents analyzed are accessible through the sources cited in the article. Due to confidentiality commitments made to research participants, the interview data cannot be shared openly. Anonymized excerpts are reported in the article and further anonymized material may be available from the authors upon request and subject to ethical restrictions.

## Footnotes

1
https://www.comune.milano.it/aree-tematiche
